# Valsalva Maneuver in the Assessment of Recurrent Cholesteatoma: A Case Report

**DOI:** 10.7759/cureus.102208

**Published:** 2026-01-24

**Authors:** Cornelius J Sauerman, Mel Corbett, Sven Geukens, Joseph P Hughes

**Affiliations:** 1 Otolaryngology - Head and Neck Surgery, University Hospital Limerick, Limerick, IRL; 2 Otolaryngology, Royal College of Surgeons in Ireland, Dublin, IRL

**Keywords:** case report, cholesteatoma, computed tomography (ct), recidivism, valsalva maneuver

## Abstract

Cholesteatoma is a benign but destructive otologic disease with a tendency to recur or be incompletely removed during primary surgical management. Patients frequently undergo long-term clinical surveillance to facilitate early detection of recurrent disease. In this case report, we describe an interesting finding in a patient with recurrent cholesteatoma. The patient was asked to perform the Valsalva maneuver, which resulted in a large amount of keratin debris being expelled into the external auditory canal. To our knowledge, there are no reports on the use of the Valsalva maneuver in the assessment of cholesteatoma recurrence.

## Introduction

Early detection and complete surgical removal of cholesteatoma minimizes complications, including hearing loss, facial nerve injury, and intracranial sequelae [1]. Otoscopy is one method of detecting cholesteatoma. CT of the temporal bone and diffusion-weighted MRI (DW-MRI) aid in diagnosis, provide information on the extent of the disease, and assist with preoperative planning of the surgical approach. Disease location, anatomical distortion from previous surgery, and clinical misdiagnosis are some reasons why cholesteatoma can be missed.

The Valsalva maneuver was first described in 1704 by Antonio Maria Valsalva. This maneuver involves a forced expiration against a closed glottis, which increases middle ear pressure through the eustachian tube [2]. While often utilized for symptomatic relief of middle ear pathology and in the assessment of tympanic membrane motility, the Valsalva maneuver has not been described in routine cholesteatoma surveillance. The Valsalva maneuver is the preferred first-line treatment in adults with chronic otitis media with effusion [3]. Using the maneuver to assess tympanic membrane compliance is a recognized examination technique [4].

## Case presentation

We present a patient in his 40s who previously underwent a left canal wall-down mastoidectomy for cholesteatoma. He was asymptomatic for 18 years and attended biannual aural microsuction in the outpatient clinic uneventfully. He presented with a four-month history of recent-onset left otorrhea and aural fullness.

**Figure 1:**
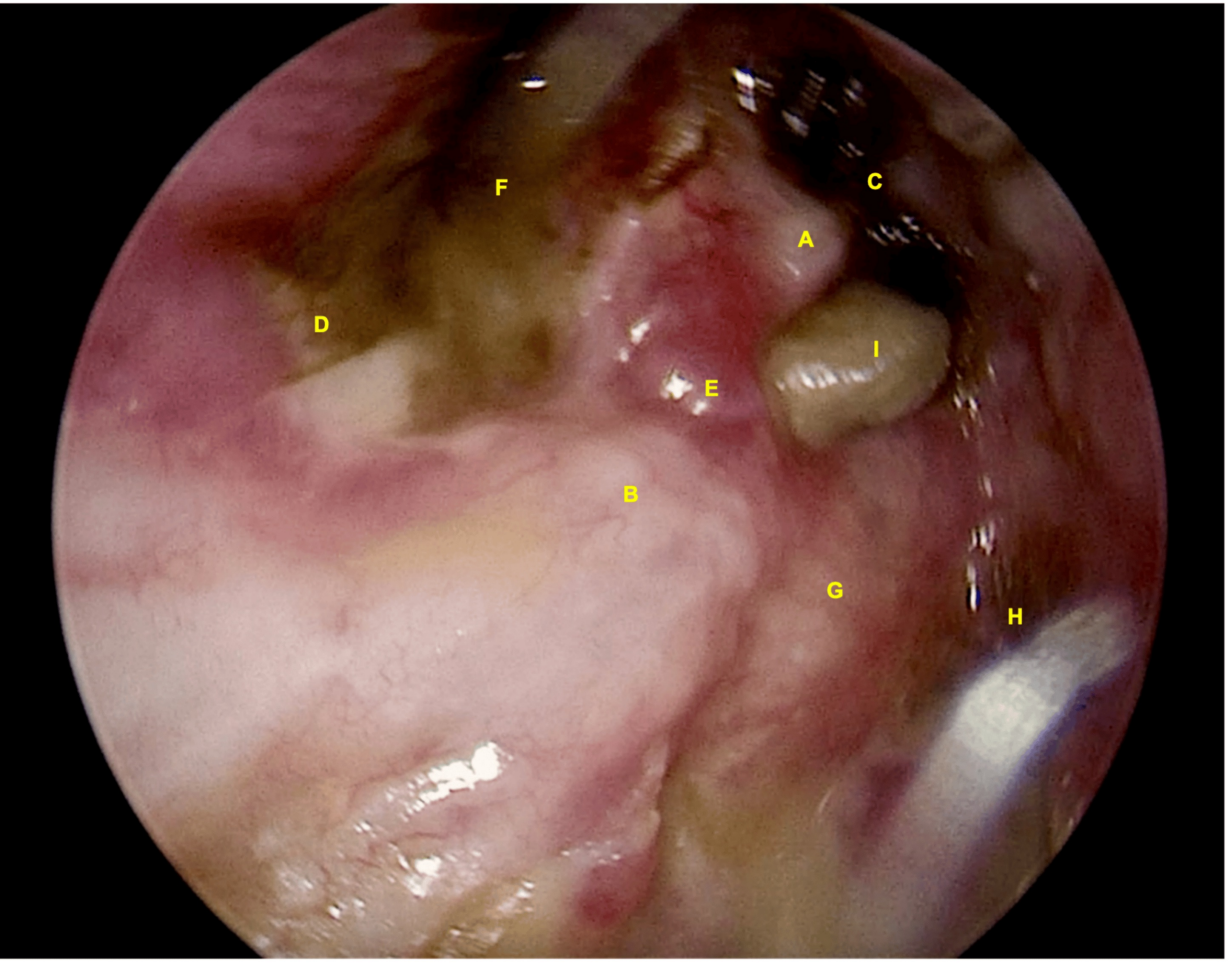
Oto-endoscopic examination (A) Malleus. (B) Facial ridge. (C) Anterior epitympanic space with crust formation. (D) Hypotympanum. (E) Posterosuperior part of the tympanic membrane. (F) Infected anterior mesotympanic part of the tympanic membrane. (G) Horizontal semicircular canal. (H) Mastoid cavity. (I). Disease protruding from the middle ear - oval window niche over the facial nerve toward the posterior epitympanum.

Otoscopy revealed a hard crust covering the neo-attic. After removal of the crust, keratin was visible. Imaging excluded a labyrinthine fistula, after which the patient was asked to perform a short, gentle Valsalva maneuver. This caused a large amount of keratin to be displaced into the external auditory canal around the head of the malleus (Figure 1, Video 1). In the video, positive pressure in the middle ear caused the keratin to move from behind the neotympanum into view. The Valsalva maneuver aided in confirming recurrent disease and evaluating its depth and extent. No symptoms or signs of a labyrinthine fistula were observed during the maneuver. This sign is most likely to be appreciated in patients who have previously undergone a canal wall-down mastoidectomy. The absence of this sign does not exclude recurrent disease.

**Video 1:** Oto-endoscopic examination Note the keratin debris being expelled into the external auditory canal during the Valsalva maneuver.

**Figure 2:**
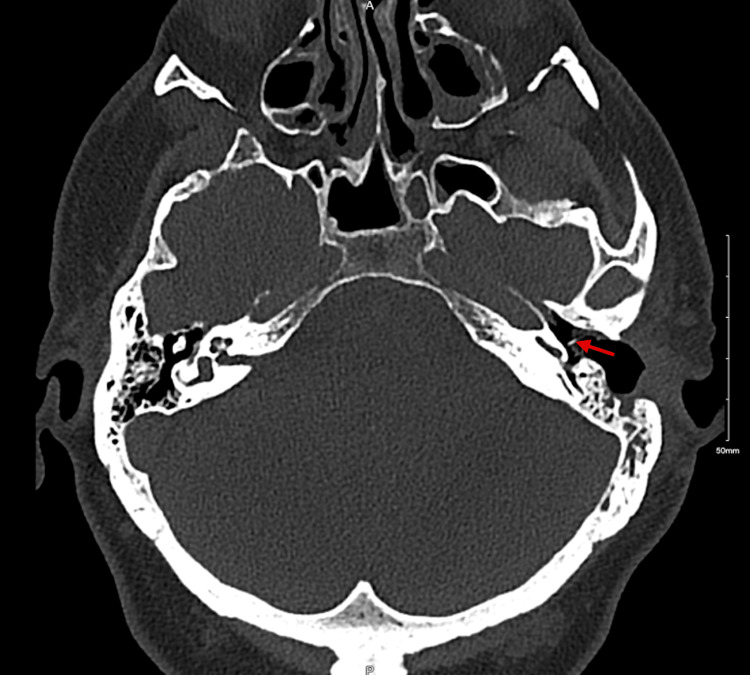
Axial section of CT temporal bone scan Axial slice demonstrating disease recurrence in the left ear with ossicular chain erosion. The right middle ear cavity appears normal.

**Figure 3:**
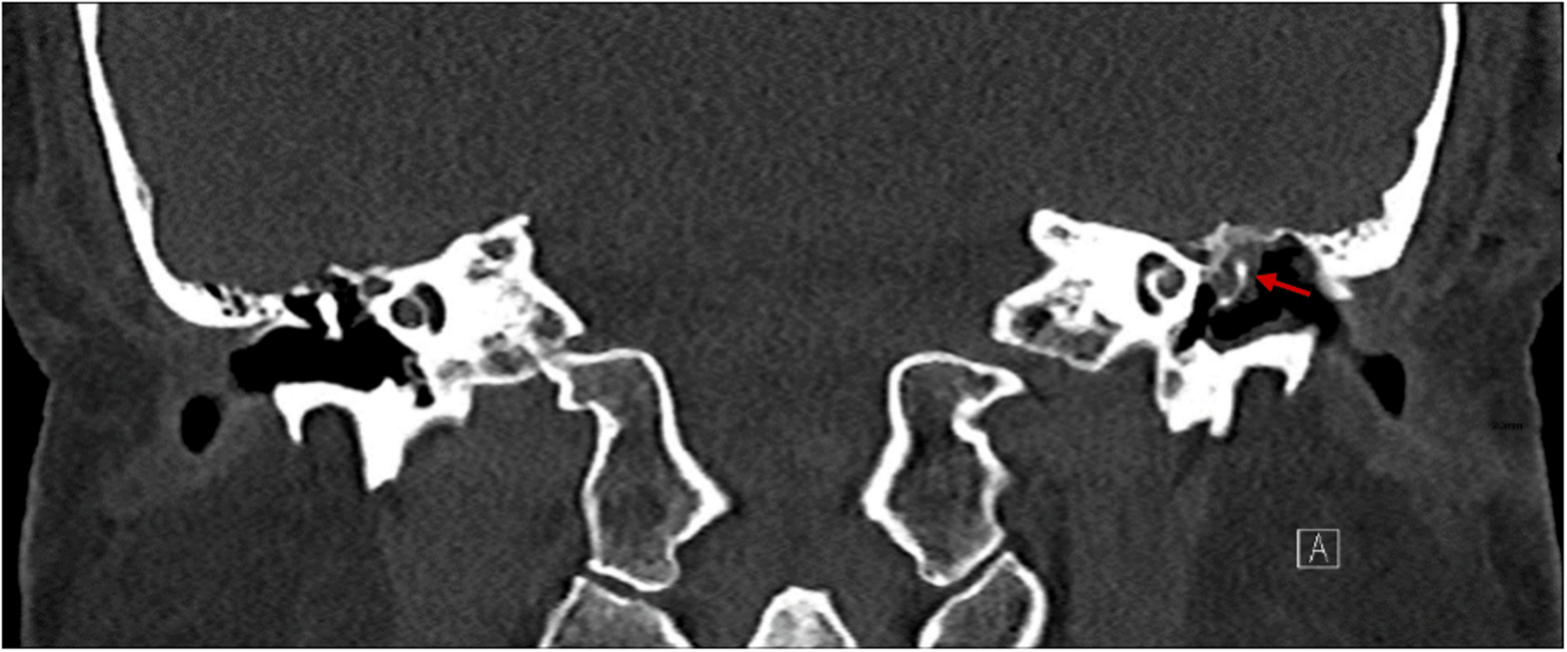
Coronal section of CT temporal bone scan Coronal view illustrating extensive middle ear ossicular chain erosion in the left ear compared to a healthy right middle ear. The tympanic segment of the facial nerve is encased by cholesteatoma.

**Figure 4:**
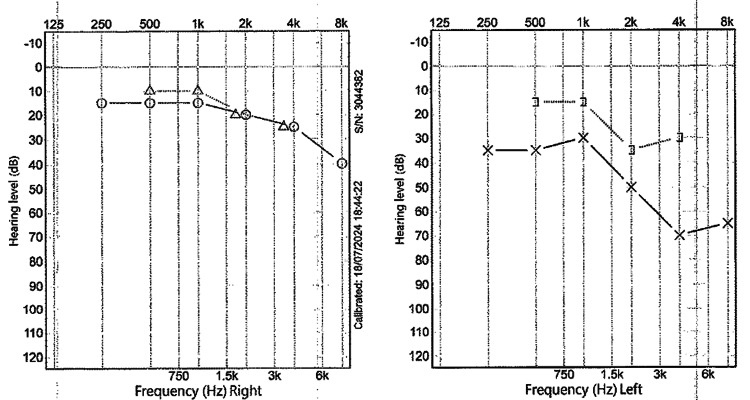
Preoperative audiogram Audiogram performed four months before revision tympanomastoidectomy. The left ear demonstrates mixed hearing loss.

**Figure 5:**
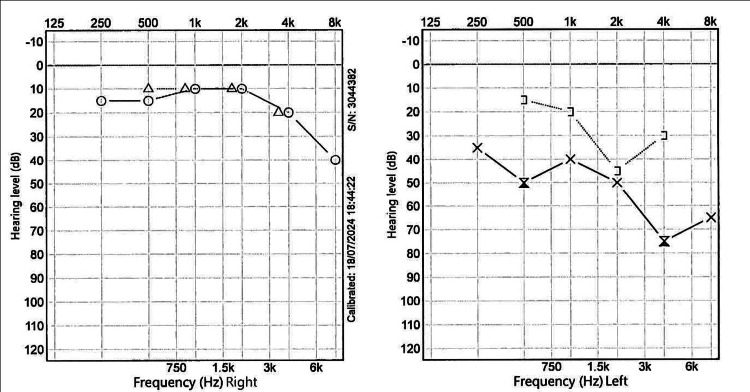
Postoperative audiogram Audiogram six months after revision tympanomastoidectomy shows thresholds similar to the preoperative evaluation.

CT of the temporal bone (Figure 2) demonstrated disease recurrence, ossicular chain erosion, and encasement of the tympanic segment of the facial nerve (Figure 3). Imaging excluded a labyrinthine fistula. Audiometry (Figure 4) illustrated mixed hearing loss. The patient underwent a revision tympanomastoidectomy. Six months postoperatively, the patient is asymptomatic and continues regular outpatient follow-up for microdebridement of the mastoid cavity. Oto-endoscopic examination shows no evidence of keratin reaccumulation and a well-epithelialized cavity. Audiometry (Figure 5) at six months postoperatively shows similar thresholds compared to the preoperative evaluation.

## Discussion

Cholesteatoma recurrence is common, approaching 40% at 15-year postoperative follow-up [5]. Multiple studies have investigated the most accurate methods to predict the risk of recurrence. Kaplan-Meier survival analysis [6], which is cost-free, has been shown to be one of the most accurate methods [7]. Several risk factors for recurrence have been identified, including pediatric disease, acquired rather than congenital etiology, left-sided disease, and previous revision surgery [5]. The surgical approach also influences recurrence risk. Canal wall-down mastoidectomies are associated with lower recurrence rates and a decreased incidence of persistent otorrhea [8]. A systematic review in 2015 hypothesized that recurrence after canal wall-down mastoidectomy is more likely due to true disease recurrence, whereas recurrence after canal wall-up mastoidectomy often results from residual disease [9]. In our patient, several of these risk factors were present. In patients with canal wall-down mastoid cavities, the endoscope allows visualization deep into retraction pockets or “around corners” [10].

Selecting the appropriate imaging modality is critical, particularly in suspected recurrence. CT of the temporal bones provides sufficient information regarding bony involvement; however, when available, non-contrast DW-MRI is the preferred modality for detecting recurrence [11]. In some cases, it has even been proposed as an alternative to revision surgery [12] and is particularly useful in patients who have undergone previous middle ear surgery, with a positive predictive value of 93% [13].

**Figure 6:**
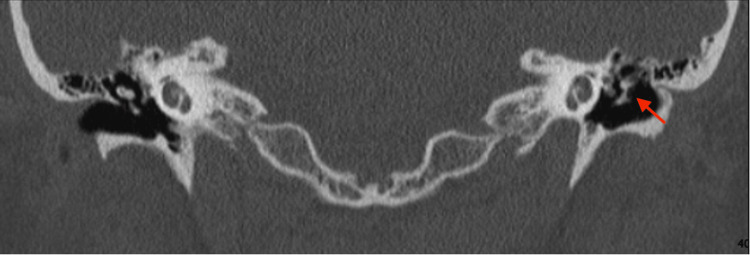
Coronal section of the CT internal auditory meatus This CT scan was performed in 2007 during the initial diagnostic workup. No erosive changes were reported.

Compared to the previous CT of the internal auditory meatus performed in 2007 (Figure 6), no erosive changes were reported. Due to long waiting lists and the urgency of the case, the patient did not undergo DW-MRI.

An important contraindication to performing the Valsalva maneuver in patients with cholesteatoma is the presence of a labyrinthine fistula. Labyrinthine fistulas result from endochondral bone loss of the labyrinth, most commonly associated with cholesteatoma [14]. Lateral semicircular canal fistulas are the most prevalent type [15]. Vertigo, dizziness, tinnitus, and disequilibrium induced by increased middle ear pressure or loud noise may indicate a labyrinthine fistula [16]. Additional contraindications for performing the Valsalva maneuver include coronary artery disease, valvular pathology, arrhythmias, and retinopathies [17]. To our knowledge, the prevalence and sensitivity of this sign in the literature have not been reported.

## Conclusions

Endoscopic evaluation of cholesteatoma has transformed our understanding of the disease and allows superior visualization and detection of recurrent cholesteatoma. In this case, endoscopic examination provided a better perspective of disease dynamics and extent compared to handheld otoscopy. Similar to endoscopic evaluation of the laryngeal inlet, respiratory maneuvers may improve appreciation of disease extent and aid diagnosis.

We propose utilizing this maneuver to assist in the diagnosis of recurrent cholesteatoma, as demonstrated in our video; however, the absence of this sign does not exclude disease. It is important to note that this maneuver should only be applied in a select group of patients after contraindications are excluded. While the maneuver may be nonspecific, this does not preclude its use in appropriate cases. With advances in imaging and novel approaches to enhance clinical assessment, earlier detection and treatment of cholesteatoma could improve patient outcomes.
